# Open questions on emergence in chemistry

**DOI:** 10.1038/s42004-022-00667-7

**Published:** 2022-04-07

**Authors:** Vanessa A. Seifert

**Affiliations:** Department of History and Philosophy of Science, University of Athens, Athens, Greece

**Keywords:** Communicating chemistry, Chemical education

## Abstract

Strong emergence is the main form of emergence that has been defended with respect to chemistry, and in particular molecular structure. Here, the author spells out this form of emergence, proposes new ways in which one can further explore the question of emergence, and explains why investigating emergence should be of interest not only to philosophers but to chemists as well.

Are chemical substances, clouds, tigers, humans, tables just the result of interactions between fundamental physical particles? Is there anything else to these things beyond the physical stuff that make them up? In response to this conundrum, emergence is the idea that there is something more to things than what we can say about them by looking only at their constituents. Emergentism was proposed as an alternative to reductionism; namely the view that everything around us is composed and completely determined by the interactions between fundamental physical particles. Reductionism became particularly popular during the highly fruitful development of physics in the 20th century. In this context, quantum mechanics and statistical mechanics became paradigmatic examples of the success of reduction as they illustrated that it is possible to describe, explain and predict the behaviour of macroscopic matter in terms of the interactions of its constitutive physical particles.

## Emergence in chemistry: the case of molecular structure

Returning to emergence, a variety of case studies from the natural sciences have been invoked in support of and against this idea, including from chemistry. In fact, chemistry is a particularly helpful case through which one can investigate and understand emergence. First, this is because chemistry has a relatively uncontested set of well-established and empirically supported theories and descriptions of phenomena. Secondly, through the formulation of quantum chemistry it has established a well-defined and explicit scientific connection to quantum mechanics, thus providing philosophers robust material that they can use in order to spell out its connection to fundamental physics. Thirdly, unlike biology, chemistry is not burdened with difficult questions around life or the nature of consciousness, thus allowing philosophers to focus on the ways in which chemical entities relate to interactions between fundamental physical particles. (Special thanks to James Ladyman for bringing this last point to my attention.)

Nevertheless, the matter of emergence in chemistry is far from settled. As philosophers of chemistry have shown, there are specific examples of chemical properties whose connection to fundamental physics cannot be straightforwardly understood in terms of either reduction or emergence. The most debated case is that of molecular structure. When one describes a molecule’s structure through quantum mechanics- i.e., by solving the relevant molecular Schrödinger equation- one cannot do so unless she presupposes certain facts about the examined structure. Specifically, one needs to apply -among other things- the Born Oppenheimer approximation which involves the assumption that the molecule has determinate nuclear positions. While this is justified scientifically by the fact that “the ratio of electronic to nuclear mass is sufficiently small”, philosophers have taken this move as an indication of a deeper problem^1^. Specifically, it has been argued that this supports the non-reducibility of chemistry. Quantum mechanics -on its own and without presupposing facts about the examined system- cannot derive a description of the system’s chemical properties.

Various views have been proposed that attempt to explain this alleged failure of reduction. The one which is of interest for the present purposes is that of emergence. For the case of molecular structure this has been most successfully spelled out by Robin Hendry who argues that molecular structure strongly emerges^2^. On this view, the reason why structure cannot be identified from quantum mechanics (i.e., without making prior assumptions about the molecule’s structure) is because its structure does not exist at the scale which is described by quantum mechanics. While structure is partially determined by how the subatomic particles interact with each other, there is something ‘over and above’ those interactions.

Does this mean that emergence posits mysterious powers that determine a molecule’s structure? No. Emergence does not posit mysterious or additional forces other than the four fundamental forces postulated by physics. It does not even deny that there is a close relation between the chemical properties and the subatomic particles of a molecule (this relation is usually called supervenience). Nevertheless, strong emergence maintains that molecular structure is not fully determined by the interactions of the molecule’s physical parts. This is because structure itself partly determines how the system’s quantum mechanical entities will behave. Put differently, the way a molecule is structured is part of the cause of how its subatomic particles interact. This idea is called downward causation as it posits a causal relation between a molecule’s structure and the interactions of its subatomic particles.

It is in this sense that molecular structure is standardly understood as emergent. And indeed, the empirical evidence that has been brought forward for its support is not to be disregarded. This evidence primarily focuses on the quantum mechanical description of isomers. It has been pointed out that even if one solved the Schrödinger equation from first principles, the relevant ground state of an isomer would in that case correspond not to a specifically observed isomeric structure but to a superposition of all its possible isomeric structures. This is taken to suggest that there is an apparent mismatch between what we empirically observe and what quantum mechanics predicts.

Strong emergence explains this by the fact that a specific isomeric structure is not reduced to the sum of interactions of the molecule’s subatomic particles but rather emerges at a different scale. However, Franklin and Seifert have offered an alternative explanation. They argue that this mismatch is an instance of the measurement problem in quantum mechanics, namely the problem that arises due to the incompatibility between unique determinate outcomes and the fact that quantum mechanics “tells us that physical systems are sometimes well described by superposition states in the basis corresponding to some observable quantities”^3^.

Based on this, Franklin and Seifert claim that structure has to be re-examined from a completely different perspective: i.e., one that takes into account different interpretations to quantum mechanics. This is because each interpretation offers a different understanding of what superpositions are and different solutions to the measurement problem. However, this does not necessitate the rejection of emergence. There are existing views about emergence that have been formulated in relation to particular interpretations to quantum mechanics^4^. So there is still room to defend emergence, though admittedly in a way that is more closely informed by how one deals with foundational problems in quantum mechanics.

## Open questions about emergence

Investigating foundational issues in quantum mechanics is not the only way to further explore chemistry’s emergence. Chemistry postulates various entities, properties and processes that go beyond atoms and molecules. As such, it offers case studies which have been so far neglected and which could be used to further understand emergence. One such example are chemical reactions. In the philosophy of chemistry, chemical reactions have mostly been examined with respect to the form of explanations they offer to chemists. Interestingly, they have not been extensively examined philosophically in terms of how they relate to fundamental physics. In part this is because of the implicit assumption that should we understand how atoms and molecules relate to their quantum mechanical constituents, then there is no need to examine how chemical reactions - as processes among atoms or molecules- relate to physics. But this is not obviously so. While chemical reactions are indeed in one sense just descriptions of chemical transformations among atoms and molecules, they can also be considered representations of processes that encompass rich and diverse information from chemistry, quantum mechanics, and thermodynamics. How do all these sciences come together and what does this mean for the relation between chemistry and physics? Raising such questions could lead to novel ways of understanding both reduction and emergence in chemistry.

Another way in which emergence in chemistry can be further explored is by investigating different and previously unexplored forms of emergence^5^. In philosophy of science, many different forms of emergence have been proposed that either have to do with how different theories or representations relate to each other, or with different ways in which entities in the world relate. Applying such understandings of emergence and investigating them from the perspective of chemistry can be beneficial to better understanding not only the relation of chemistry to physics but also the relations of other sciences as well.

## Conclusion: should chemists care about the question of emergence?

A standard challenge raised against philosophical questions such as that of emergence, is that they have no bearing onto the work done in chemistry. And indeed it seems that whatever philosophers say about the nature of molecular structure or chemistry’s relation to quantum physics, little will change in how chemists go about with their work. To some extent this is how things should be. But it is worth pointing out that there is value for chemists to think of emergence and of other philosophical ideas around chemistry. This is for three reasons. First, philosophical investigations around science can provide novel and deeper insight into how science is done and what science tells us about the world. For example, investigating reduction and emergence in chemistry can be the means to appreciate the conceptual intricacies that are involved in correctly delineating chemistry’s relation with the other sciences. Secondly, philosophical questions can be invoked to motivate or support the investigation of novel research questions in science. For example, the philosophical implications of the Born-Oppenheimer approximation (mentioned above) can be used to justify scientific studies that focus on whether and how quantum mechanics describes chemical phenomena without applying this approximation. Thirdly, what is often disregarded but is particularly important is the impact of philosophy on chemical education. It has been argued that chemical education can greatly benefit from an informed philosophical analysis of chemistry^6^. In the context of inter-theory relations this is quite evident: background assumptions regarding chemistry’s relation to fundamental physics can greatly alter the manner in which one teaches the nature of chemical entities and properties.
